# UnpadStat Design: Portable Potentiostat for Electrochemical Sensing Measurements Using Screen Printed Carbon Electrode

**DOI:** 10.3390/mi14020268

**Published:** 2023-01-20

**Authors:** Riyanto Setiyono, Tias Febriana Hanifa Lestari, Anni Anggraeni, Yeni Wahyuni Hartati, Husein Hernadi Bahti

**Affiliations:** 1 Department of Chemistry, Faculty of Mathematics and Natural Sciences, Padjadjaran University, Jatinangor 45363, Indonesia; 2 Department of Electrical Engineering, Faculty of Engineering, Langlangbuana University, Bandung 40162, Indonesia

**Keywords:** potentiostat, portable, SPCE, touch screen, SD card

## Abstract

In this research a portable potentiostat was built for electrochemical sensing measurements with three electrodes, specifically SPCEs. The circuit uses a microcontroller as the main controller to manage all activities, starting from adjusting the input voltage for the SPCEs, setting measurement parameters, measuring the resulting current, displaying graphics on the touch screen, sending data to the computer via the USB port, and connecting to the SD card. Measurements and errors with cyclic voltammetry techniques have been compared with commercial potentiostats. The measurement results on a dummy circuit and commercial SPCEs have an accuracy of more than 90% compared to commercial potentiostats. In addition, measurement data can also be saved to an SD card in .CSV format for further purposes.

## 1. Introduction

Several studies on potentiostats have recently been carried out. Graphical reading of measurement results from the biosensor still uses other tools such as computers, laptops, or smartphones. Potentiostats are key devices in modern electrochemical research related to redox chemical reactions and other chemical phenomena. In this study, Arduino was used, which was designed for cyclic voltammetric characterization and used to study redox reactions that occur at the electrode-electrolyte interface. The design is simple, easy to operate, and has low cost and good performance. Graphical reading is done with the help of a computer via a USB connection [1,2]. Some potentiostats that use interfacing with computers are potentiostat/galvanostat for thin-film battery characterization [3], potentiostat for teaching electrochemistry and instrumentation [4], CheapStat: potentiostat for analytical and educational applications [5], DStat: potentiostat for electroanalysis and integration [6], potentiostat with two microprocessors for electrochemical biosensors [7], and potentiostat for electrochemical biosensing measurement system [8]. Apart from these, other potentiostat studies using a smartphone connection wirelessly for graph reading include wireless potentiostat for electrochemical detection [9], KAUSTat: wireless potentiostat for electrochemical measurements [10], wireless potentiostat for multivariate data processing [11], and potentiostat wireless for electrochemical biosensor for point-of-care diagnostics [12]. Apart from the studies above, there is also a patent on a portable potentiostat that uses a battery and has its own display, namely the Portable instrument for field ready electrochemical experimentation [13].

The aim of this research is to design and implement a potentiostat circuit that can capture redox reactions in a typical three-electrode electrochemical sensor measurement system, namely the Working Electrode (WE), Counter Electrode (CE), and Reference Electrode (RE). In this case the sensor used is based on a screen printed carbon electrode (SPCE). SPCE is a type of SPE which has many advantages, namely requiring few samples, relatively cheap, easy to carry, and easy to use [14]. The designed circuit can be used to detect small current changes in SPCE-based sensors. A comparison of several microcontroller-based potentiostats that have been made, including this research (UnpadStat), can be seen in Table 1. UnpadStat has several advantages, namely being portable because it uses a battery, having its own display for setting parameters, storing data on an SD card for further analysis, and it can be connected to a computer for more detailed observation of measurement data.

## 2. UnpadStat Design

**Figure 1:**
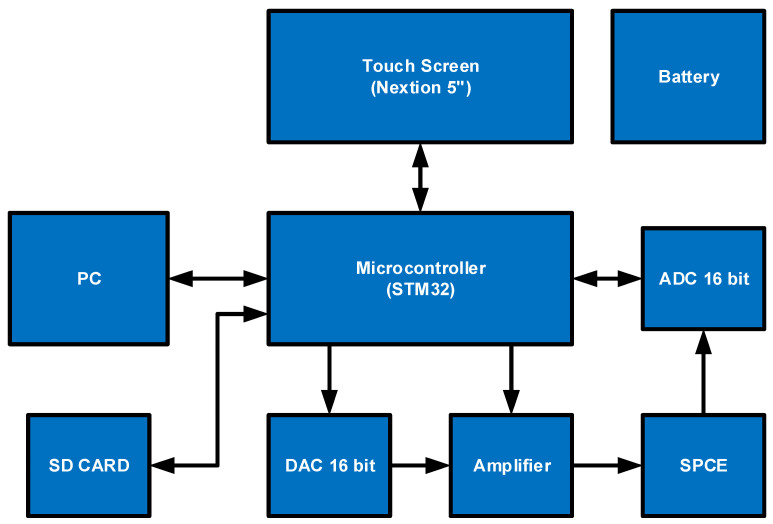
UnpadStat block diagram.

The complete block diagram of the circuit can be seen in Figure 1. The input section is an SPCE-based sensor, which is an important gate where electrochemical reactions occur. The output section is divided into three, namely touch screen (Nextion 5 inch), computer, and SD card, where each of these outputs has different parameters. The most important part is the STM32-based microcontroller which manages the supporting components such as the 16-bit digital to analog converter (DAC), amplifier, and 16-bit analog to digital converter (ADC).

The circuit design is divided into several parts to make it easier to analyze the potentiostat circuit.

A. DAC

**Figure 2:**
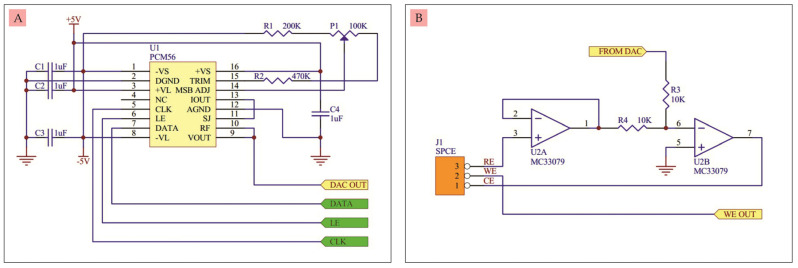
(**A**) 16-bit DAC circuit, (**B**) Buffer and differential amplifier circuit.

DAC serves to convert digital signals into analog signals or voltages. The DAC used here was an IC with the PCM56P type which is often used in audio. This IC has several advantages including stability, low noise, 16-bit resolution, small current, wide symmetrical supply voltage, and requires few additional components to work. The output voltage is also wide and can be adjusted from −3 V to +2.9999 V [15]. The output voltage regulation is carried out through three ports, namely the DATA, LE, and CLK ports. These three ports must be connected to the microcontroller. The complete DAC circuit can be seen in Figure 2A. Capacitors C1, C2, C3, and C4 are used to stabilize the voltage. Resistors R1, R2, and Potentiometer P1 are used to calibrate the output voltage.

B. Buffer circuit and differential amplifier

The buffer circuit and differential amplifier were built from two op-amps of type MC33079. This IC has low noise, high frequency stability, wide symmetrical supply voltage, and small current [16]. The buffer circuit and differential amplifier can be seen in Figure 2B. The buffer circuit is needed so that the voltage generated by the Reference Electrode (RE) of the SPCE is stable and does not burden the SPCE. The voltage from the buffer is then compared with the voltage coming from the DAC using a differential amplifier circuit. The difference in voltage is fed back to the Counter Electrode (CE). By providing a voltage sweep on the DAC, it will produce a varying output current at the Working Electrode (WE).

C. Current to voltage converter circuit

**Figure 3:**
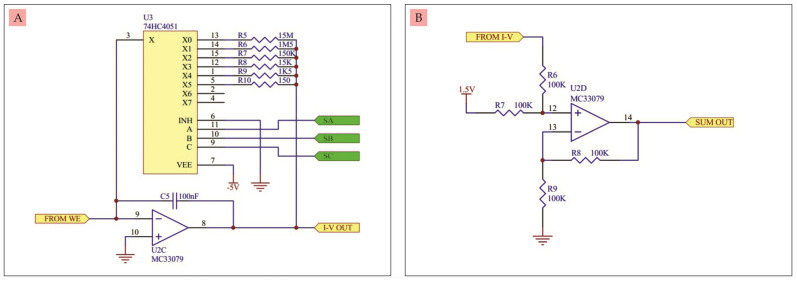
(**A**) Current-to-voltage converter circuit, (**B**) Positive voltage summing amplifier circuit.

The circuit in Figure 3A was built from an MC33079 op-amp and an 8-channel electronic switch 74HC4051 [17]. MC33079 serves to convert the current coming out of the WE and into a voltage at the output of the op-amp. The voltage is directly proportional to the incoming current. Its voltage gain is determined by either resistors R5, R6, R7, R8, R9, or R10. The selection of resistors connected to the circuit is regulated by the IC 74HC4051 via the SA, SB, and SC ports. Meanwhile C5 serves to stabilize the voltage on the selected multiplier resistor. This resistor selection is useful for selecting a measurement scale ranging from tens of nano amperes to milli amperes.

D. Positive voltage summing amplifier circuit

The circuit in Figure 3B was built from an op-amp MC33079, which functions to add up the negative voltage from the current-to-voltage converter into a positive voltage in increments of 1.5 V. This circuit is also called a voltage level shifter. The purpose of using this circuit is so that there is no negative voltage at the output.

E. ADC

**Figure 4:**
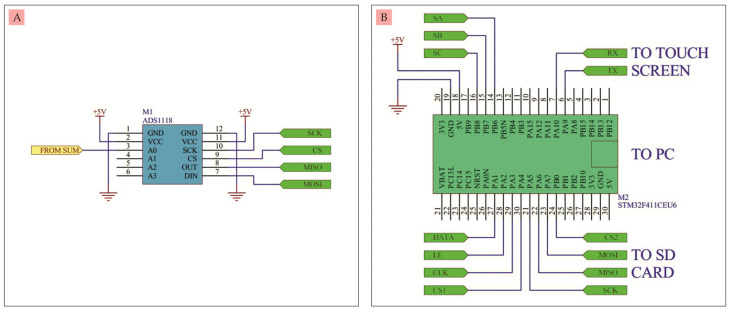
(**A**) 16-bit ADC circuit, (**B**) Microcontroller.

ADC serves to convert analog signals or voltages into digital signals. The ADC used here was a module in which there is an ADS1118 IC which has a 16-bit resolution and four inputs, namely A0, A1, A2, and A3 [18]. Only one input was used in this circuit, namely input A0. The ADC circuit and ports for serial communication via SPI with a microcontroller can be seen in Figure 4A. The ADC output from this circuit can then be processed digitally by the microcontroller.

F. Microcontroller

The microcontroller functions to control the flow of data starting from adjusting the DAC output voltage, adjusting the speed of the voltage sweep, reading the voltage generated by the ADC which is a read current conversion, displaying all data in the form of graphs and numbers to the touch screen, storing data on the SD card, and/or transmitting data to the computer via the USB port. The microcontroller used was the STM32F411CEU6 module, which can process 32-bit data at a speed of 100 MHz [19], as can be seen in Figure 4B. This microcontroller is programmed in advance using STM32duino to run all stages of input and output settings, so that the potentiostat can work according toits function.

G. Touch screen

The touch screen is used to display all input and output data in the form of graphs. In addition, potentiostat setting data and measurement steps can be displayed and can be adjusted using this touch screen. The touch screen used was Nextion 5 inch with type NX8048K050 [20]. Similar to the microcontroller, this touch screen must be programmed first using NextionEditor so that it can work as desired.

H. SD card

The SD card functions to store all experimental data, starting from input data, output data, and setting data. The data is saved using the comma separated values (.CSV) format which is plain text that contains a list of data. For one experiment, a maximum storage capacity of 12 Kbyte is required, so if the SD card used is 8 Gigabyte, it can accommodate up to 600,000 experimental data.

I. Battery

There are 3 lithium ion batteries used in this circuit with a voltage of 3.7 V and a capacity of 2500 mA which are installed in parallel. The circuit requires a symmetrical voltage of ±5 V so that a voltage conversion from a single voltage to a symmetrical voltage is required via the XL6007 DC to DV converter. The battery can supply the circuit for more than 14 h continuously.

## 3. Results and Discussion

A. Casing design

**Figure 5:**
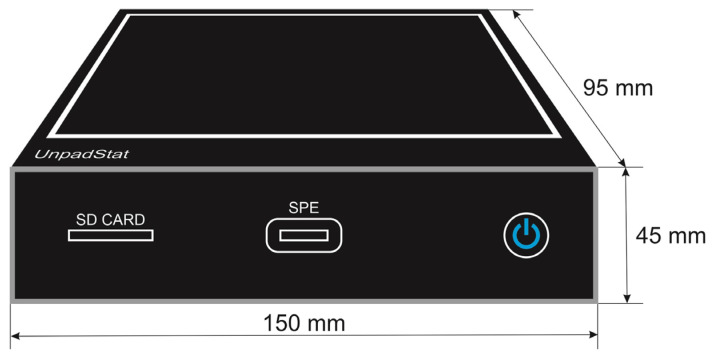
Casing design.

The casing was designed using black acrylic to accommodate the circuit, battery and 5-inch touch screen, making it a compact and portable device. The casing design with dimensions of 150 × 95 × 45 mm can be seen in Figure 5.

B. Specification

Specifications from UnpadStat and its comparison with the patent entitled Portable instrument for field ready electrochemical experimentation can be seen in Table 2.

C. Testing

**Figure 6:**
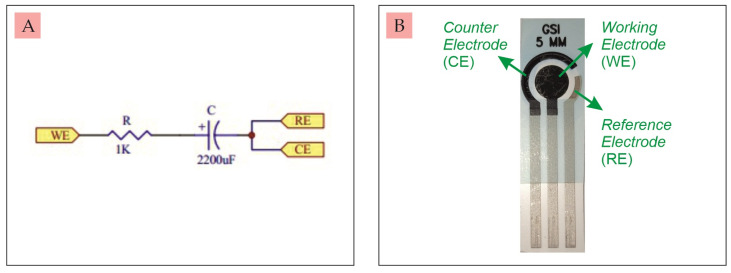
(**A**) Dummy circuit, (**B**) Commercial SPCE.

The UnpadStat test was carried out using the cyclic voltammetry (CV) technique for two experiments. The first and second experiments were analyzed via a touch screen and connected to a computer using Microsoft Visual C# 2019 software. The measurement results displayed on the touch screen and on the computer were exactly the same, because the measurement data was sent simultaneously to both. The first experiment was a current measurement using a dummy circuit [3] consisting of one resistor (R) and one capacitor (C), as shown in Figure 6A. The second experiment was a current measurement using a commercial SPCE in Figure 6B.

**Figure 7:**
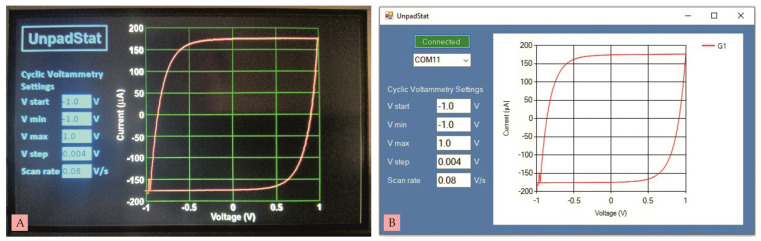
(**A**) CV measurement on a dummy circuit using Nextion, (**B**) CV measurement on a dummy circuit using a computer.

The results of CV measurements on the dummy circuit can be seen in Figure 7A,B. Current graphs that are read tend to be stable. The voltage supplied by the potentiostat was from −1 V to +1 V at a speed of 80 mV/s with a resolution of 4 mV. The current generated after the process of charging and discharging the capacitor remained at a positive peak current of 176.64 µA and a negative peak current of −176.44 µA.

**Figure 8:**
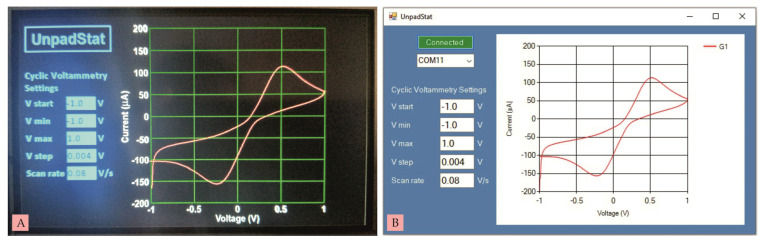
(**A**) CV measurement on commercial SPCE using Nextion, (**B**) CV measurement on commercial SPCE using computer.

The results of CV measurements on SPCE that had been dripped with 10 mM Ferricianide K_3_[FE(CN)_6_] solution can be seen in Figure 8A,B. The voltage given was the same as the previous test, namely from −1 V to +1 V at a speed of 80 mV/s with a resolution of 4 mV. The anodic peak is around 0.5 V and the cathodic peak is around −0.25 V. The resulting anodic peak current (Ipa) is 113.13 µA and the cathodic peak current (Ipc) is 156.72 µA.

**Figure 9:**
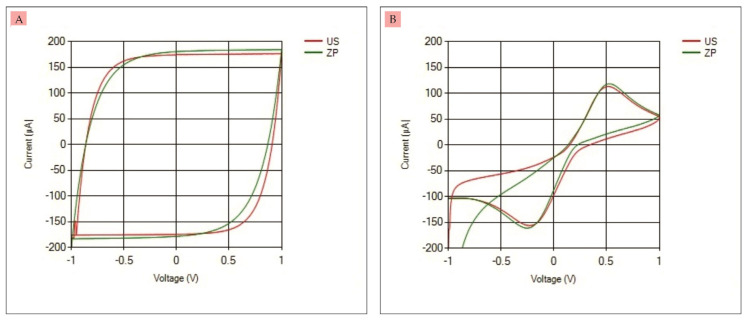
(**A**) CV measurement on dummy circuit using UnpadStat and commercial potentiostat, (**B**) CV measurement on commercial SPCE using UnpadStat and commercial potentiostat.

Furthermore, the results of CV measurements in the first and second experiments were compared with commercial potentiostats (Ana Pot X4—EIS—ZP). The results can be seen in Figure 9A,B, where the graphs in red are the results of UnpadStat measurements and the graphs in green are the results of the commercial potentiostat measurements. The accuracy of the CV measurement results on the dummy circuit and commercial SPCE can be seen in Table 3. The accuracy on the dummy circuit for Ipa is 95.88% and for Ipc is 96.12%, while the accuracy on the commercial SPCE for Ipa is 95.66% and for Ipc is 97.19%.

**Figure 10:**
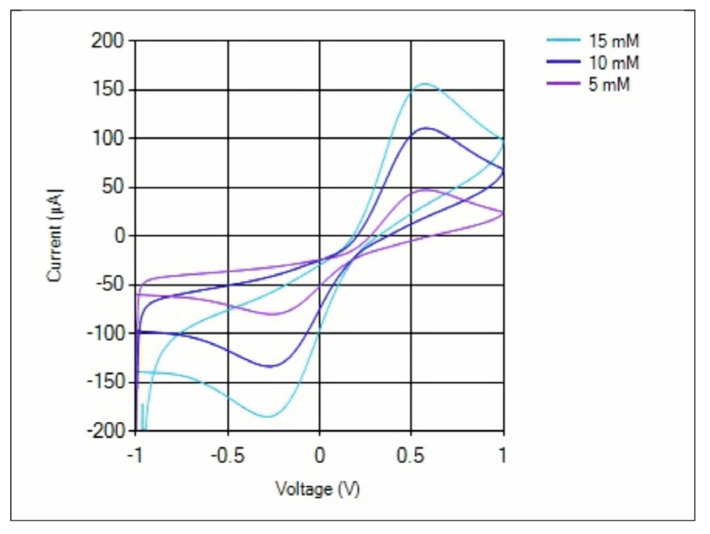
CV measurement on SPCE which had been dripped with 15 mM, 10 mM, and 5 mM Ferricianide K_3_[FE(CN)_6_] solution.

The last measurement was the CV measurement on SPCE which had been dripped with 15 mM, 10 mM, and 5 mM Ferricianide K_3_[FE(CN)_6_] solution. The voltage supplied was from −1 V to +1 V at a speed of 50 mV/s with a resolution of 4 mV. The measurement results can be seen in Figure 10. The higher the concentration of the solution, the higher the Ipa and Ipc.

## 4. Conclusions

In this study a potentiostat (UnpadStat) designed for three-electrode electrochemical sensing measurements worked well. The resulting graphics are clear without significant noise, both on the touch screen and on the computer. UnpadStat’s accuracy is quite high on the 4th measurement scale (15 KΩ multiplier resistor), with a voltage sweep from −1 V to +1 V at a speed of 80 mV/s with a resolution of 4 mV. The accuracy obtained when compared with a commercial potentiostat for measuring dummy circuit and commercial SPCE is more than 90%. Data can also be stored on an SD card in .CSV format of 12 kilobytes, so using an SD card with a capacity of 8 gigabytes can store up to 600,000 trial data.

## Figures and Tables

**Table 1:** Comparison of the main features of the reported potentiostats.

No	Microcontroller	Measurement Methode	Communication Interfaces	Own Display	SD Card/ Flashdisk	Ref.
1	Arduino Mega2560	CV	USB	-	Flashdisk	[1]
2	Teensy 3.2	CV, LSV, chronoamperometry, chronocoulometry	USB	-	-	[2]
3	PIC16F1459	CV	USB	-	-	[3]
4	Arduino Uno	CV, chronoamperometry	USB	-	-	[4]
5	AVR XMEGA	CV, LSV, SWV	USB	-	-	[5]
6	AVR XMEGA	CV, SWV, DPV	USB	-	-	[6]
7	C8051F005	CV, LSV, DPV, amperometry, potentiometry	USB	-	SD card	[7]
8	Microcontroller 32 bit	CV, DPV	USB	-	-	[8]
9	RFduino	CV, SWV, chronoamperometry, potentiometry	Bluetooth	-	-	[9]
10	Unreported	CV	Bluetooth	-	-	[10]
11	AVR XMEGA	CV, LSV, SWV	Bluetooth	-	-	[11]
12	PIC16F685	CV	Microphone	-	-	[12]
13	Arduino	CV, ASV, LSV, PSV	USB	Touch screen	on board	[13]
14	STM32F411CEU6	CV	USB	Touch screen	SD card	This work

**Table 2:** Comparison of UnpadStat specifications with a patent entitled portable instrument for field ready electrochemical experimentation.

No	Feature	UnpadStat	Portable Instrument for Field Ready Electrochemical Experimentation
1	Battery life	14 h include touch screen	52 h
2	Sweep rate limit	±10–100 mV/s	±10–400 mV/s
3	Sweep value limit	±3 V	±2.5 V
4	Voltage step	±0.1–100 mV	unreported
5	Variable gain	6 gain levels	14 gain levels automated
6	Linear dynamic range	±100 nA–10 mA	±600 pA–1 mA
7	Storage	up to 32 Gb removable	32 Gb removable

**Table 3:** Comparison of CV measurement results.

	Dummy Circuit	SPCE Commercial
UnpadStat		
Ipa (µA)	176.64	113.13
Ipc (µA)	−176.44	−156.72
Ana Pot X4—EIS—ZP		
Ipa (µA)	184.23	118.26
Ipc (µA)	−183.56	−161.25
UnpadStat accuracy relative to Ana Pot X4—EIS—ZP		
Ipa (%)	95.88	95.66
Ipc (%)	96.12	97.19

## Data Availability

Not applicable.
